# Heart Failure Prescribing Quality at Discharge from a Critical Care Unit in Egypt: The Impact of Multidisciplinary Care

**DOI:** 10.3390/pharmacy8030159

**Published:** 2020-09-01

**Authors:** Seif El Hadidi, Naglaa Samir Bazan, Stephen Byrne, Ebtissam Darweesh, Margaret Bermingham

**Affiliations:** 1 Pharmaceutical Care Research Group, School of Pharmacy, University College Cork, 30 Cork, Ireland; Seif.Yehia@fue.edu.eg (S.E.H.); Stephen.Byrne@ucc.ie (S.B.); 2 Faculty of Pharmaceutical Sciences and Pharmaceutical Industries, Future University in Egypt, Cairo 11835, Egypt; naglaabazan@cu.edu.eg (N.S.B.); edrweesh@fue.edu.eg (E.D.); 3 Critical Care Medicine Department, Cairo University Hospitals, Cairo University, Giza 12511, Egypt

**Keywords:** pharmacist, heart failure, multidisciplinary care, guideline adherence, guideline-directed medical therapy, Egypt

## Abstract

Discharge prescriptions for heart failure (HF) patients may not adhere to the clinical practice guidelines. This study aimed to assess the impact of the clinical pharmacist as a member of a multidisciplinary team on the quality of prescribing to HF patients at discharge from a Critical Care Unit (CCU) in Egypt. This was a retrospective cohort study of HF patients discharged from the CCU between January 2013 and December 2017. Guideline Adherence Index (GAI-3) was used to assess guideline-directed prescribing at discharge. Multidisciplinary care was introduced to the CCU on 1 January 2016. The study included 284 HF patients, mean (±SD) age 66.7 ± 11.5 years, 53.2% male. Heart failure with reduced ejection fraction affected 100 patients (35.2%). At discharge, loop diuretics were prescribed to 85.2% of patients; mineralocorticoid receptor antagonists to 54.9%; angiotensin-converting enzyme inhibitors/angiotensin receptor blockers to 51.4%; and β-blockers to 29.9%. Population Guideline Adherence Index (GAI-3) was 45.5%. High-GAI was prescribed to 136 patients (47.9%). Patients with High-GAI were younger; less affected by chronic kidney disease and had fewer comorbidities than those without High-GAI. Prescription of β-blocker increased (24.1% vs. 38.6%, *p* < 0.001) and digoxin utilization decreased (34.7% vs. 23.7%, *p* < 0.049) after the introduction of the multidisciplinary care. The inclusion of a clinical pharmacist in the multidisciplinary care team may have a role in optimizing the prescribing of HF guideline-directed therapies at discharge from this setting.

## 1. Introduction

The prevalence of heart failure (HF) is estimated to be 2% of the total adult population in developed countries, rising to greater than 10% among people aged 70 years and older [1]. In Europe, HF exacerbation is the cause of more than 1 million hospitalizations annually [2] and rehospitalization is common among patients with HF following their initial discharge [3]. European data demonstrate that 12-month all-cause mortality rates for hospitalized and ambulatory HF patients are 17% and 7%, respectively [4]. The exact prevalence of HF in Egypt is unknown; however, evidence suggests that the disease emerges a decade younger in the Middle East and North Africa (MENA) region than in Europe [5,6]. The available MENA data suggest that HF patients are more likely to be severely symptomatic due to delayed diagnosis or late presentation to healthcare settings in comparison to patients from Europe and North America [7,8]. While recent data for Egypt are not available, the International Congestive Heart Failure registry showed that for HF patients, one-year all-cause mortality in the Middle East was 9%, and in Africa was 34% [8].

In HF, the international clinical practice guidelines strongly recommend the prescription of HF guideline-directed medical therapies (GDMT) at target doses [9,10,11,12,13]. Guideline-led prescribing is associated with clinical benefits including improved survival rates; reduced frequency of hospitalization and rehospitalization; reduced length of stay; reduction in adverse cardiovascular events; and decreased use of mechanical ventilation [10,11,14]. In the BIOSTAT-CHF and QUALIFY studies, the optimization of HF GDMT and the prescription of ≥50% of target doses of these agents demonstrated short- and long-term benefits in patient survival and rehospitalization outcomes [14,15].

Patient hospitalization presents an opportunity to implement HF GDMT in a monitored setting. However, studies show that discharge therapeutic plans for HF patients are often not adherent to the clinical practice guidelines [16,17]. In one long-term registry, prescription rates of HF GDMT at hospital discharge were 83.6% for diuretics, 77% for angiotensin-converting enzyme inhibitors/angiotensin receptor blockers (ACEI/ARB), 71.8% for β-blockers, 55.3% for mineralocorticoid receptor blockers (MRA), and 3.2% for ivabradine [17]. Two studies by Gilstrap and colleagues identified reasons for the omission of HF GDMT during hospitalization or at discharge including renal dysfunction and hypotension [18,19].

Many HF patients are first cared for in the critical care setting and the prescribing decisions made in this setting may influence future prescribing and medical stability. In Egypt, many patients admitted to the Critical Care Medicine Department affiliated to Cairo University, Faculty of Medicine, Qasr Al-Ainy are discharged directly from critical care to their homes and decisions about long-term HF medications are made at this timepoint. However, little is known about the quality of HF prescribing at discharge from critical care settings. The acutely ill HF population represents a challenge for prescribers as these patients are often older, suffering from multiple acute comorbidities, prescribed appropriate and potentially inappropriate polypharmacy, and are more likely to experience contraindications to therapies [3,17,20]. Therefore, discharge prescribing may not be optimized in this population [3,17].

Multidisciplinary care is considered the gold standard model for the seamless delivery of guideline-directed HF management [11,21] and implementation of multidisciplinary care can improve the transition of care and reduce rehospitalization rates by up to 30% [22,23]. The inclusion of clinical pharmacists in HF multidisciplinary care teams has been shown to optimize guideline-led prescribing during and after hospitalization [24]. However, there are no reports of multidisciplinary HF care involving a clinical pharmacist in critical care settings. 

This study aimed to assess the impact of the clinical pharmacist as a member of a multidisciplinary team on the quality of prescribing to HF patients at discharge from a Critical Care Unit (CCU) in Egypt. 

## 2. Materials and Methods

This is a retrospective cohort study of HF patients hospitalized in the 53-bed CCU of Cairo University Hospitals between 1 January 2013 and 31 December 2017. Ethics approval was granted by the Research and Ethics Committee of Future University in Egypt, Cairo, Egypt (registration number REC-FPSPI-9/56). Permission to conduct the research using the electronic database of the department was granted by the Management Board of the Critical Care Medicine Department of Cairo University Hospitals prior to data collection. As this was a retrospective study and data were anonymized at source, patient consent was not required for the study. The study is reported according to the Strengthening the Reporting of Observational Studies in Epidemiology (STROBE) guidelines [25].

Patients were included if they were ≥18 years on the date of admission, had a diagnosis of HF, had an electronic record of discharge medications, and were discharged from the CCU during the study period. Patients were excluded from the study if they died during admission or if they remained an inpatient on the final day of the study period. The diagnosis and type of HF were based on data recorded in the patient’s electronic medical record. According to the European Society of Cardiology (ESC) guidelines that were in place during the study period, heart failure with reduced ejection fraction (HFrEF) was defined as an ejection fraction ≤40% [10]. Data accessed in the patient’s electronic medical record included age, gender, admission date, discharge date, presenting complaint, comorbidities, laboratory investigations, and medical investigations. The following information on discharge medications was also accessed in the electronic medical records: drug name, dose, and frequency. Hyperpolypharmacy was defined as the prescription of ≥10 regular medications per day. Hyperpolypharmacy was used as a measure of medication burden as this population is prescribed a high number of medications, both for HF and for comorbidities [26]. The median dose of loop diuretic was calculated to reflect HF severity [27].

The ESC Guidelines for the Diagnosis and Treatment of Acute and Chronic Heart Failure 2012 were used in this study as they are the guidelines that were in place for most of the study timeframe [10]. The primary outcome of the study was to assess HF guideline-led prescribing at discharge using the Guideline Adherence Index (GAI-3) [28], the adjusted GAI-3 [29], and the GAI-based target dose [30]. The GAI-3 was calculated as the ratio of the medications prescribed to the medications that should theoretically have been prescribed according to the guidelines. These medications are agents within the following classes: ACEI/ARB, evidence-based β-blockers (EBBB) and MRA [10,28]. The EBBBs in HF are bisoprolol, carvedilol, metoprolol succinate, and nebivolol [10]. The adjusted GAI-3 considered the patient’s contraindications to these therapies as outlined in the guidelines (Table 1) [10,29,31]. The GAI-based target dose was calculated as a prescription of ≥50% of the guideline-recommended target dose of each of the three GAI medications (Table 1) [10,30]. The study population was then subdivided into those with High-GAI management; that is the prescription of ≥2 of the GAI-3 medications and those with Low-GAI management; that is the prescription of ≤1 GAI-3 medication [31].

Multidisciplinary care was provided to all patients in the CCU from 1 January 2016 onwards. The multidisciplinary care team consisted of critical care physicians and clinical pharmacists. The clinical pharmacy team consisted of (i) a clinical pharmacy supervisor with 15 years’ experience as a critical care pharmacist in the CCU, who held a Master of Science and a Doctor of Philosophy degree in Clinical Pharmacy from Cairo University; (ii) a pharmacist who held a PharmD from the Faculty of Pharmacy, Cairo University—this is a two-year post-graduate program after a five-year bachelor of Pharmaceutical Sciences or Bachelor of Clinical Pharmacy degree; and (iii) four pharmacists who held a Bachelor of Clinical Pharmacy degree from the Faculty of Pharmacy, Cairo University. The clinical pharmacists were available daily at the CCU from 8 am to 3 pm except on Fridays. The role of the clinical pharmacist was (i) to participate in the daily morning physician-led ward round to provide prescribing recommendations; (ii) to perform medication review and medication reconciliation on admission in order to identify drug therapy problems; and (iii) to provide a drug information service to the critical care physicians. 

Normally distributed data were presented as mean ± standard deviation (SD), and non-normal data as the median and the interquartile range. Categorical data were presented as frequencies and percentages. Comparisons between (i) patients with High-GAI and Low-GAI based management and (ii) patients receiving care before and after the introduction of multidisciplinary care were conducted using independent Student’s *t*-test or Mann–Whitney U test for continuous data and Chi-square or Fisher’s exact test for categorical data. All tests were two-tailed, and a *p*-value < 0.05 was regarded as statistically significant. 

Univariable logistic regression analysis was performed, and a multivariable logistic regression model was developed in order to determine the clinical factors associated with High-GAI achievement. The multivariable logistic regression model included the variables that were considered clinically relevant and variables that demonstrated a significant difference in the comparison between High-GAI and Low-GAI populations. The adjusted odds ratios (OR) and 95% confidence intervals (CI) of the multivariable analysis were reported. Data were analyzed using SPSS^®^ (IBM SPSS Statistics for Windows, Version 22.0., IBM Corp., Armonk, NY, USA). 

## 3. Results

### 3.1. Baseline Profile and Characteristics of Heart Failure Patients

Data were available for 284 patients who had a documented diagnosis of HF. The mean ± SD age of the patients was 66.7 ± 11.5 years, and 53.2% were male. Ejection fraction was available for 220 patients, and the mean ± SD ejection fraction was 45.1% ± 16.7%. HFrEF affected 100 patients (35.2%). Coronary artery disease was the HF etiology in 132 patients (46.5%), and acute coronary syndrome was the main presenting complaint in 81 patients (28.5%). The mean ± SD number of comorbidities was 5.2 ± 2.4; with hypertension (*n* = 140, 49.3%), diabetes (*n* = 130, 45.8%) and atrial fibrillation (*n* = 109, 38.4%) the most frequently occurring comorbidities (Table 2).

### 3.2. Prescribing to the Heart Failure Population

At discharge, the mean ± SD number of daily medications was 9.1 ± 2.5 (Table 2). Fourteen patients (4.9%) were not prescribed any HF medications. Prescription rates for the three GDMT were ACEI/ARB, *n* = 146 (51.4%); EBBB, *n* = 85 (29.9%); and MRA, *n* = 156 (54.9%). A combination of two GDMT was prescribed to 94 (33.1%) patients, and all three medicines were prescribed to 42 (14.8%) patients. Prescription of ≥50% of the guideline-recommended target doses of ACEI/ARB, EBBB, and MRA was achieved in 40 (14.1%), 21 (7.4%) and 145 (51.5%) patients, respectively. Although not a GAI-3 medication, the most frequently prescribed HF medication was loop diuretics (*n* = 242, 85.2%), with 45 patients (15.8%) prescribed a loop diuretic as their only HF medication and 43 (15.2%) patients prescribed two or more loop diuretic agents at discharge. 

No patient experienced a contraindication to ACEI/ARB or MRA. At least one contraindication to EBBB therapy was present in 70 (24.6%) patients, 23 (8.1%) having a second or third-degree AV-block, and 47 (16.5%) having asthma. Of these 70 patients, 21 (30.0%) were prescribed an EBBB at discharge.

Population mean GAI-3 was 45.5%, and adjusted GAI-3 was 51.3%. The GAI-3 target dose was 24.3%. 

### 3.3. High-GAI and Low-GAI Achievement

**Figure 1:**
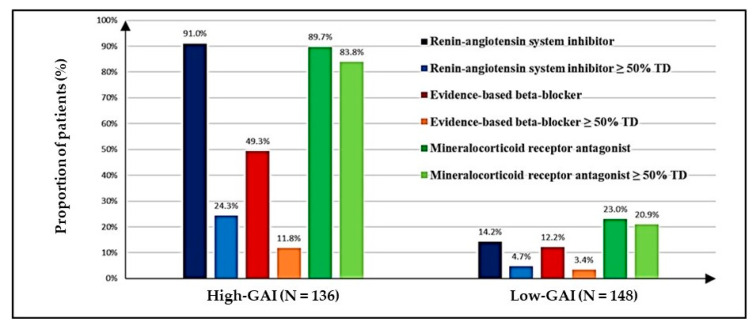
Prescription of guideline-directed medical therapies and achievement of ≥50% target dose for each medication class, presented as High-Guideline Adherence Index (High-GAI) population and Low-Guideline Adherence Index (Low-GAI) population. The proportion of patients prescribed each medication class was compared between High-GAI and Low-GAI populations. This comparison for each of the three GAI medicines was statistically significant (*p* < 0.001). The proportion of patients prescribed ≥50% target dose of each medication class was compared between High-GAI and Low-GAI populations. This comparison for each of the three GAI medicines was statistically significant (*p* < 0.001). The target dose is defined in Table 1. GAI: guideline adherence index; TD: target dose.

High-GAI based management was achieved in 136 patients (47.9%). These High-GAI patients were younger (62.6 ± 10.7 vs. 70.5 ± 11.0 years, *p* < 0.001); more likely to be male (65.4% vs. 41.9%, *p* < 0.001); more likely to have HFrEF (49.3% vs. 22.3%, *p* < 0.001); had fewer comorbidities (4.9 ± 2.3 vs. 5.6 ± 2.5, *p* = 0.017); and were less likely to have chronic kidney disease (22.1% vs. 33.8%, *p* = 0.028) than those patients with Low-GAI. The prescription of recommended target doses of ACEI/ARB, EBBB, and MRA was significantly higher in the High-GAI cohort than the Low-GAI cohort (Figure 1). Higher median doses of loop diuretics were prescribed to the Low-GAI cohort in comparison to the median doses prescribed to the HF patients with a High-GAI based management; however, the difference did not reach significance, 40 mg/day [60–120 mg/day] vs. 20 mg/day [40–80 mg/day], *p* = 0.731. 

### 3.4. Contribution of Multidisciplinary Care

There were few differences in demographics or comorbidities between HF patients receiving the routine care of the critical care physician (*n* = 170) and those receiving multidisciplinary care (*n* = 114) (Supplementary Table S1). The rate of atrial fibrillation was higher among those in the multidisciplinary care arm and patients in the multidisciplinary care arm were more likely to achieve a heart rate <70 bpm and to have elevated blood urea nitrogen. There was no difference in the median dose of loop diuretics between the two groups. Medications prescribed to the two cohorts are described in Table 3. Patients who received multidisciplinary care were more likely to be prescribed an EBBB (38.6% vs. 24.1%, *p* < 0.001) and were less likely to be prescribed digoxin (23.7% vs. 34.7%, *p* = 0.049) than those receiving physician-only care. 

### 3.5. Logistic Regression Analysis

The multivariable model included the following variables: age, sex, the number of comorbidities, HFrEF, blood urea nitrogen >20 mg/dL, serum creatinine >2.5 mg/dL, and prescription of ivabradine. In the multivariable logistic regression analysis, the clinical factors associated with High-GAI management were age (adjusted OR 0.93, 95% CI 0.90–0.96), serum creatinine >2.5 mg/dL (adjusted OR 0.55, 95% CI 0.37–0.82) and HFrEF (adjusted OR 1.16, 95% CI 1.05–1.25). The model estimation correctness was 78.8% and Nagelkerke’s R2 = 0.44.

## 4. Discussion

The present study is the first assessment of HF guideline-led prescribing at discharge from a critical care setting. At discharge, the mean guideline adherence was 45.5%, adjusted GAI-3 was 51.3%, and when adjusted for the achievement of ≥50% target dose, it was 24.3%. After the implementation of multidisciplinary care involving a pharmacist, the adherence level did not significantly change, however, some changes in the prescribing patterns of individual HF medications were observed in particular an increase in the prescription of EBBB.

The prescription rates and the level of High-GAI achievement reported in this study are lower than those reported in QUALIFY. QUALIFY is an international registry that included Egyptian HF patients recently discharged primarily from cardiology settings rather than CCU settings. [32] A recent systematic review found that in studies published from 2005 to 2016, GAI-3 ranged from 14% to 95%, with a mean GAI-3 of 62.9% [31]. While the GAI-3 reported here is lower than this international mean [31], it is comparable to recently reported GAI-3 in Brazil (41%) [33] and Korea (43%) [34], however, these studies were conducted in different settings. The study population here is more acutely ill than others reported in the GAI literature and this represents a serious challenge to prescribers in comparison to ambulatory HF populations or HF populations hospitalized in non-critical care settings [31]. 

The differences reported here between patients with High-GAI and those with Low-GAI reflect the adverse impact of age and multimorbidity on guideline adherence. Patients with Low-GAI were older, had a higher comorbidity burden and higher serum creatinine levels than High-GAI patients. The adjusted GAI-3 considers the contraindications to therapy listed in the guidelines. However, in the present study, adjusting for these contraindications had little effect on correcting guideline adherence levels. Meanwhile, it is possible that prescribers take other considerations into account when prescribing GDMT. For instance, almost 30% of the population experienced chronic kidney disease, and these patients were significantly less likely to be prescribed High-GAI than patients with normal kidney function. This suggests that reduced renal function may represent a barrier to the appropriate prescription of ACEI/ARB and MRA in acute-care settings [15,35].

In the present study, the high prescription rate of loop diuretics and MRAs reflects the acutely ill status of the patients admitted to the critical care setting. Given that the medications were recorded at discharge this high rate of diuretic use may also indicate prescribers’ preference for short-term symptom relief over longer-term disease-modifying interventions. Additionally, the high rate of loop diuretics prescribed and low rate of EBBB may suggest that these patients remain congested. The prescription of a fixed-dose formulation containing furosemide and spironolactone contributed to higher target dose achievement among patients prescribed MRA than the other GDMT. This fixed-dose combination is available on the Egyptian market at a low price. These affordable products may enhance patient compliance and persistence, and prescribers may be influenced by such practical considerations. Furthermore, a high incidence of diuretic resistance has been reported among Egyptian patients, and adjunct medications such as thiazide diuretic metolazone are not commonly included in the hospital formularies [6,36]. The inaccessibility of adjunct diuretics such as metolazone may also have contributed to the unexpectedly high rate of prescription of two or more loop diuretics.

The low rate of target dose achievement reported here may reflect the critical care setting from which the patients are being discharged, the prescribers’ focus on acute illness rather than long-term outcomes and an assumption that doses may be titrated upwards in an ambulatory cardiology setting. For instance, 53% of patients in the “BIOSTAT-CHF” study required a 12-week stepwise approach to reach ≥50% of the recommended target dose [15]. This highlights the need for introducing patient education and counseling sessions for HF patients at discharge. Such a service would advise patients of the importance of follow-up, the need for medication optimization over the coming months, and the importance of medication adherence.

There is no doubt that HF management is complex and multifaceted. As a consequence, guidelines recommend a multidisciplinary approach to the optimal and seamless delivery of HF care [3,11]. Egyptian reports before 2015 show high rates of digoxin use and underutilization of EBBB [6,37]. In the present study, the implementation of multidisciplinary care significantly increased EBBB prescription and significantly decreased digoxin prescription. The prescribing changes reported here indicate improved adherence to the most recent ESC guidelines [11]. However, the overall GAI-3 and the proportion of patients achieving High-GAI-based management did not significantly increase with the implementation of multidisciplinary care. The pharmacists in the multidisciplinary team could make a recommendation about patient medications but had no authority to implement the changes to inpatient or discharge prescriptions. Unfortunately, the acceptance rate of interventions was not available in this study. Others have reported on the phenomenon of physicians’ encroachment and their reluctance to alter a colleague’s prescription despite appropriate recommendations made by clinical pharmacists [38,39,40]. This may adversely affect the influence on prescribing quality of multidisciplinary care. 

Some limitations of this study must be acknowledged. The study is retrospective, single-centered, and includes only the patients’ discharge medications, therefore medications trialed on an inpatient basis could not be assessed. As it was not possible to randomize and in order to reduce the risk of selection bias, all HF patients discharged before the introduction of the service were compared to all HF patients discharged after the introduction of the multidisciplinary HF service. Unfortunately, with this retrospective design, a causal relationship could not be assessed.

## 5. Conclusions

This study is the first to consider HF guideline adherence on discharge from a critical care unit and the impact of multidisciplinary care in Egypt, or indeed, any low-middle income country. It identifies some inconsistencies between guideline-recommended HF prescribing and the current routine practice and also highlights the potential for greater pharmacist involvement in the HF multidisciplinary team.

## Figures and Tables

**Table 1:** Guideline-directed medical therapies, their contraindications, and target doses as described in the European Society of Cardiology Guidelines for the Diagnosis and Treatment of Acute and Chronic Heart Failure 2012 [10].

Medication Class	Contraindications	Agents	100% Target Daily Dose
ACE inhibitor/Angiotensin Receptor Blocker	History of angioedema Known bilateral renal artery stenosis Pregnancy/risk of pregnancy	Captopril Enalapril Lisinopril Ramipril Candesartan Losartan Valsartan	150 mg 20 mg 20 mg 10 mg 32 mg 150 mg 320 mg
Evidence-based β-blockers	Second- or third-degree Atrio-ventricular block Asthma	Bisoprolol Carvedilol Nebivolol	10 mg 50 mg 10 mg
Mineralocorticoid Receptor Antagonists	Eplerenone use with strong cytochrome inhibitors	Spironolactone Eplerenone	50 mg 50 mg

Agents listed are those agents from each class, prescribed to one or more patients in the study population. ACE, angiotensin-converting enzyme.

**Table 2:** Baseline characteristics and medications profile of the total population, patients prescribed High-GAI, and patients prescribed Low-GAI, N = 284 patients.

N = 284 Patients	Total Population	High-GAI	Low-GAI	*p*-Value
	(N = 284)	(N = 136)	(N = 148)	
**Clinical profile**				
Age (years)	66.7 ± 11.5	62.6 ± 10.7	70.5 ± 11.0	<0.001
Male	151 (53.2)	89 (65.4)	62 (41.9)	<0.001
Mean arterial pressure (mmHg)	94.9 ± 17.6	93.3 ± 19	96.3 ± 16.2	0.436
Heart rate (bpm)	86.2 ± 22.0	87.9 ± 21.6	84.6 ± 22.3	0.701
HFrEF	100 (35.2)	67 (49.3)	33 (22.3)	<0.001
Hypertension	140 (49.3)	69 (50.7)	71 (48.0)	0.313
Atrial fibrillation	109 (38.4)	48 (35.3)	61 (41.2)	0.541
Coronary artery disease	132 (46.5)	69 (50.7)	63 (42.6)	0.376
Diabetes	130 (45.8)	60 (44.1)	70 (47.3)	0.132
Chronic kidney disease	80 (28.2)	30 (22.1)	50 (33.8)	0.028
Asthma/COPD	64 (22.5)	34 (25.0)	30 (20.3)	0.812
Number of comorbidities	5.2 ± 2.4	4.9 ± 2.3	5.6 ± 2.5	0.017
**Clinical Status at Discharge**				
Low blood pressure (<90/60 mmHg)	9 (3.5)	8 (6.7)	1 (0.7)	0.011
High blood pressure (>140/90 mmHg)	88 (34.6)	34 (28.6)	54 (40.0)	0.214
Heart rate ≤ 70 bpm	107 (37.7)	46 (33.8)	61 (41.2)	0.333
Heart rate ≥ 100 bpm	57 (20.1)	28 (25.5)	29 (24.0)	0.412
Hyperkalemia (K^+^ > 5.0 mg/dL)	9 (3.2)	3 (2.2)	6 (4.1)	0.877
High blood urea nitrogen (>20 mg/dL)	153 (53.9)	63 (46.3)	90 (60.8)	<0.01
High serum creatinine (>2.5 mg/dL)	31 (10.9)	7 (5.1)	24 (16.2)	<0.01
Length of stay	9.8 ± 6.9	9.3 ± 7.4	10.3 ± 6.5	0.049
**Discharge Medications Profile**				
ACEI/ARB	146 (51.4)	125 (91.9)	21 (14.2)	0.011
EBBB	85 (29.9%)	67 (49.3)	18 (12.2%)	0.214
MRA	156 (54.9)	122 (89.7)	34 (23.0)	0.333
Digoxin	86 (30.3)	48 (35.3)	38 (25.7)	0.412
Loop Diuretics	242 (85.2)	120 (88.2)	122 (82.4)	0.877
Ivabradine	31 (10.9)	21 (15.2)	10 (6.8)	<0.01
Regular medications	9.1 ± 2.5	9.3 ± 2.3	8.9 ± 2.6	0.049
Hyperpolypharmacy	121 (43.7)	59 (43.4)	62 (41.9)	0.011
Device-based therapy *	38 (13.4)	19 (14.0)	19 (12.8)	0.214
**Major Prescribing Patterns at Discharge**				
Loop diuretic as monotherapy	45 (15.8)	-	45 (30.4)	-
ACEI/ARB + β-blocker	56 (19.7)	56 (41.7)	-	-
ACEI/ARB + MRA	110 (38.7)	110 (80.9)	-	-
Loop diuretic + ACEI/ARB	123 (43.3)	108 (79.4)	15 (10.1)	<0.01
Loop diuretic + MRA	146 (51.4)	114 (83.8)	32 (21.6)	<0.01
Loop diuretic + MRA + Digoxin	54 (19.1)	43 (31.6)	11 (7.4)	0.021
Loop diuretic + ACEI/ARB + MRA	102 (35.9)	102 (75.0)	-	-

Comparisons were made between Heart Failure patients with High-GAI and Low-GAI. Categorical variables are expressed as frequencies and percentages. Continuous variables are expressed as mean ± standard deviation; * Device-based therapy: implantable cardiac defibrillator, cardiac resynchronization therapy, or left ventricular assistance device; ACEI/ARB, angiotensin-converting enzyme inhibitor or angiotensin receptor blocker; COPD, chronic obstructive pulmonary disease; EBBB, evidence-based β-blocker; GAI, Guideline Adherence Index; HFrEF, heart failure with reduced ejection fraction; K^+^, serum potassium; MAP, mean arterial blood pressure; MRA, mineralocorticoid receptor antagonist.

**Table 3:** Prescribing at discharge for patients receiving routine care and patients receiving multidisciplinary care, N = 284 patients.

N = 284 Patients	Routine Care ^§^ (N = 170)	Multidisciplinary Care ^§^ (N = 114)	*p*-Value
**Discharge Medications Profile**			
ACEI/ARB	91 (53.5)	55 (48.2)	0.345
ACEI/ARB ≥ 50% Target dose	25 (14.7)	15 (13.2)	0.456
EBBB	41 (24.1)	44 (38.6)	<0.001
EBBB ≥ 50% Target dose	9 (5.3)	12 (10.5)	0.218
MRA	99 (58.2)	57 (50.0)	0.546
MRA ≥ 50% Target dose	93 (54.7)	52 (45.6)	0.617
Digoxin	59 (34.7)	27 (23.7)	0.049
Loop diuretic	149 (87.6)	93 (81.6)	0.341
Dual loop diuretics	19 (11.2)	23 (20.2)	0.032
Ivabradine	18 (10.6)	13 (11.4)	0.421
Regular medications	9.0 ± 2.4	9.3 ± 2.6	0.784
Hyperpolypharmacy	71 (41.8)	50 (43.9)	0.435
**Discharge Guideline Adherence Indices**			
GAI-3 (%)	45.2	45.7	0.598
Adjusted GAI-3 (%)	50.0	52.6	0.854
GAI-Target dose (%)	25.0	23.0	0.349
High-GAI	81 (47.6)	55 (48.2)	0.881

Comparisons were made between heart failure care provided before and after the implementation of a clinical pharmacy service at the critical care unit. Categorical variables are expressed as frequencies and percentages. Continuous variables are expressed as mean ± standard deviation. ^§^ Routine care refers to the medical care provided by the critical care physician only while multidisciplinary care refers to the medical care provided by the critical care physician and clinical pharmacist; ACEI/ARB, angiotensin-converting enzyme inhibitor or angiotensin receptor blocker; CCB, calcium channel blocker; EBBB, evidence-based β-blocker; GAI, guideline adherence index; MRA, mineralocorticoid receptor antagonist.

## Supplementary Materials

The following are available online at https://www.mdpi.com/2226-4787/8/3/159/s1, Table S1: Baseline profile of patients receiving routine care and patients receiving multidisciplinary care, N = 284 patients.
