# Supplementary material for: Heart Failure Prescribing Quality at Discharge from a Critical Care Unit in Egypt: The Impact of Multidisciplinary Care
Source: Pharmacy (Basel). 2020 Sep 1;8(3):159. doi: 10.3390/pharmacy8030159 (PMC7558601; doi:10.3390/pharmacy8030159)
Supplement: Supplementary file 1 [file pharmacy-08-00159-s001.pdf]

Supplementary Table S1. Baseline profile of patients receiving routine care and patients receiving multidisciplinary care, N = 284 patients.

N = 284 Patients	Routine Care § (N = 170)	Multidisciplinary Care § (N = 114)	*p*-Value
**Clinical Profile**			
Age (years)	66.8 ± 11.7	66.6 ± 11.4	0.841
Gender (male)	90 (52.9%)	61 (53.5%)	0.933
Mean arterial pressure (mmHg)	94.9 ± 15.0	94.9 ± 21.7	0.971
Heart rate (bpm)	89.1 ± 22.7	79.8 ± 18.9	<0.001
HFrEF	58 (45%)	42 (46.2%)	0.864
Hypertension	79 (46.5%)	61 (53.5%)	0.255
Atrial fibrillation	39 (22.9%)	70 (61.4%)	<0.001
Coronary artery disease	72 (42.4%)	60 (52.6%)	0.091
Diabetes	71 (41.8%)	59 (51.8%)	0.101
Chronic kidney disease	44 (25.9%)	36 (31.6%)	0.303
Asthma/COPD	29 (17.1)	35 (30.7)	0.071
Number of Comorbidities	5.1 ± 2.4	5.4 ± 2.5	0.485
**Clinical Status at Discharge**			
Low blood pressure (<90/60 mmHg)	4 (2.4%)	5 (5.7%)	0.187
High blood pressure (>140/90 mmHg)	59 (35.5%)	29 (33%)	0.685
Heart rate < 70 bpm	31 (18.2)	56 (49.1)	<0.001
Heart rate > 100 bpm	33 (19.4)	4 (3.5)	<0.001
Hyperkalaemia (K^+ > 5.0 mg/dl)	0 (0.0)	9 (7.9)	0.103
High blood urea nitrogen (>20 mg/dl)	74 (43.5)	79 (69.3)	<0.001
High serum creatinine (> 2.5 mg/dl)	12 (7.1)	19 (16.7)	0.199
Length of stay	9.6 ± 6.4	10.2 ± 7.6	0.491

Comparisons were made between heart failure care provided before and after the implementation of a clinical pharmacy service at the critical care unit. Categorical variables are expressed as frequencies and percentages. Continuous variables are expressed as mean ± standard deviation. § Routine care refers to the medical care provided by the critical care physician only while multidisciplinary care refers to the medical care provided by the critical care physician and clinical pharmacist. bpm, beats per minute; COPD, chronic obstructive pulmonary disease; HFrEF, heart failure with reduced ejection fraction; K^+, serum potassium.
